# Interaction of Peptide Aptamers with Prion Protein Central Domain Promotes α-Cleavage of PrP^C^

**DOI:** 10.1007/s12035-018-0944-9

**Published:** 2018-02-19

**Authors:** Erica Corda, Xiaotang Du, Su Yeon Shim, Antonia N. Klein, Jessica Siltberg-Liberles, Sabine Gilch

**Affiliations:** 1 Department of Ecosystem and Public Health, Calgary Prion Research Unit, Faculty of Veterinary Medicine and Hotchkiss Brain Institute, University of Calgary, Calgary, Canada; 2 Department of Molecular Biology, University of Wyoming, Laramie, USA; 3 Department of Biological Sciences, Biomolecular Sciences Institute, Florida International University, Miami, USA

**Keywords:** Prion protein PrP, PrP α-cleavage, Prion disease, Peptide aptamer, Neurodegenerative disease, Treatment

## Abstract

Prion diseases are infectious and fatal neurodegenerative diseases affecting humans and animals. Transmission is possible within and between species with zoonotic potential. Currently, no prophylaxis or treatment exists. Prions are composed of the misfolded isoform PrP^Sc^ of the cellular prion protein PrP^C^. Expression of PrP^C^ is a prerequisite for prion infection, and conformational conversion of PrP^C^ is induced upon its direct interaction with PrP^Sc^. Inhibition of this interaction can abrogate prion propagation, and we have previously established peptide aptamers (PAs) binding to PrP^C^ as new anti-prion compounds. Here, we mapped the interaction site of PA8 in PrP and modeled the complex in silico to design targeted mutations in PA8 which presumably enhance binding properties. Using these PA8 variants, we could improve PA-mediated inhibition of PrP^Sc^ replication and de novo infection of neuronal cells. Furthermore, we demonstrate that binding of PA8 and its variants increases PrP^C^ α-cleavage and interferes with its internalization. This gives rise to high levels of the membrane-anchored PrP-C1 fragment, a transdominant negative inhibitor of prion replication. PA8 and its variants interact with PrP^C^ at its central and most highly conserved domain, a region which is crucial for prion conversion and facilitates toxic signaling of Aβ oligomers characteristic for Alzheimer’s disease. Our strategy allows for the first time to induce α-cleavage, which occurs within this central domain, independent of targeting the responsible protease. Therefore, interaction of PAs with PrP^C^ and enhancement of α-cleavage represent mechanisms that can be beneficial for the treatment of prion and other neurodegenerative diseases.

**Electronic supplementary material:**

The online version of this article (10.1007/s12035-018-0944-9) contains supplementary material, which is available to authorized users.

## Introduction

The cellular prion protein PrP^C^ is a glycosylphosphatidylinositol (GPI)-anchored plasma membrane protein most abundantly expressed in neurons [1–4]. It consists of a flexible N-terminal portion and a globular C-terminal domain, which is posttranslationally modified by the addition of one or two N-linked carbohydrate chains and a disulfide bond [3, 5, 6]. At the cell surface, PrP^C^ is located to detergent-resistant microdomains or lipid rafts [7–9]. Although the PrP^C^ function remains enigmatic, a huge body of evidence suggests its involvement in signal transduction, Cu^2+^ binding through an N-terminal octapeptide repeat region and neuroprotective activity [10–15]. PrP^C^ undergoes physiological proteolytic processing N-terminal to a central hydrophobic domain, producing the soluble N1 and the membrane-anchored C1 fragments upon α-cleavage at amino acid 110/111 [7, 16, 17], while PrP^C^ β-cleavage is induced upon oxidative stress and occurs more N-terminal around amino acid 90, producing N2 and C2 fragments [18–20]. The responsible protease mediating PrP^C^ α-cleavage also termed αPrPase [21] is still under debate. While some studies demonstrate involvement of a disintegrin and metalloproteinase 10 (ADAM10) in constitutive α-cleavage or ADAM17 upon stimulation, other reports argue against a major role of these ADAM proteases [22–26].

PrP^C^ can undergo a posttranslational conformational switch into an aggregation-prone and protease-resistant infectious isoform designated PrP^Sc^. PrP^Sc^ is the main component of prions, proteinaceous infectious particles that cause fatal neurodegenerative diseases, for example Creutzfeldt-Jakob disease (CJD) in humans, scrapie in sheep and goats or bovine spongiform encephalopathy (BSE) in cattle. Prion diseases can occur sporadic, familial or acquired by infection. They are characterized by spongiosis, astrogliosis, and accumulation of PrP^Sc^ in the brains of affected individuals [27–32]. According to the seeded nucleation model, the interaction between PrP^C^ with PrP^Sc^ seeds is required for autocatalytic prion propagation and triggers the conformational switch of PrP^C^ from a mainly α-helical conformation into PrP^Sc^ which is highly enriched in β-sheets [33, 34].

Despite intensive research, no successful treatment or prevention strategies for prion diseases have been identified. Approaches to inhibit prion accumulation include altering the trafficking of PrP^C^ in order to avoid its localisation to proposed cellular compartments of conversion, inducing the degradation of PrP^Sc^, or preventing the PrP^C^-PrP^Sc^ interaction utilizing molecules such as peptides, antibodies, or peptide aptamers [35–37]. Another option would be to stimulate the α-cleavage of PrP^C^. The C1 fragment cannot be converted into PrP^Sc^ as it is shorter than proteinase K-resistant PrP^Sc^ [3], and moreover, the C1 fragment is a dominant negative inhibitor of prion conversion [38, 39]. However, this possibility is hampered by the fact that it has not yet been convincingly elucidated which protease mediates the α-cleavage of PrP^C^.

We have previously identified peptide aptamers as a new class of anti-prion compounds. Peptide aptamers are combinatorial peptides displayed by a scaffold protein such as the *E. coli* thioredoxin A (trxA). This confers conformational stability and high binding affinity for the target protein to the peptide moiety [40, 41]. We described peptide aptamers (PAs) based on the trxA backbone, selected for interaction with mature PrP (amino acids 23–231) and demonstrated that these molecules which were expressed and purified from *E. coli* or overexpressed in the secretory pathway of persistently prion infected N2a cells and modified by the addition of C-terminal subcellular targeting signals interfered with PrP^Sc^ propagation [42, 43].

The goal of our current study was to improve the anti-prion effect of our PAs by enhancing their binding affinity for PrP^C^. We mapped the binding sites of PAs to PrP^C^ and identified PA8 to bind to amino acids 100–120 (PrP100–120), a site which covers PrP’s most conserved hydrophobic domain [44, 45] as well as large parts of the PrP neurotoxic peptide. To improve the binding affinity, we performed in silico modeling studies of the PrP100–120-PA8 complex and identified three amino acids which could be replaced with specific residues to strengthen the interaction. Based on these calculations, we produced eight mutants of PA8 with one amino acid substitution each. Three out of these eight mutants had superior effects in reducing PrP^Sc^ levels in our initial screening compared to the original PA8 and were analyzed in more detail. Their anti-prion effect was independent of the prion strain used for infection. Moreover, all PAs had a strong prophylactic effect when used to prevent new infection of 3F4-N2a cells. Mechanistically, we found that PA binding to PrP100–120 enhanced the α-cleavage of PrP^C^ and increased both the total amount and the cell surface levels of the C1 fragment, most likely upon interference with PrP internalization.

In summary, we improved the anti-prion effects of PA8 and identified new molecules that impair PrP^Sc^ propagation by two mechanisms: (i) the binding to the PrP hydrophobic domain, which presumably interferes with PrP^C^-PrP^Sc^ interaction and/or disturbs initial steps of PrP^C^ refolding, and (ii) the stimulation of α-cleavage, resulting in high levels of the C1 fragment which is a transdominant-negative inhibitor of prion conversion. This is the first study to demonstrate enhancement of PrP^C^ α-cleavage by interaction of an anti-prion molecule with the hydrophobic domain. Overall, we suggest that these mechanisms can have an impact on treatment not only of prion diseases but also for application to other neurodegenerative diseases that involve toxic interaction with the PrP^C^ central domain or benefit from its increased α-processing.

## Methods

### Mapping of Peptide Aptamer Binding Site by Yeast-2-Hybrid

Using site-directed mutagenesis, stop codons were introduced into pGBKT7-PrP90–231 at codons 120, 150, and 180 to result in N- and C-terminally truncated versions of PrP. These constructs were co-transformed with pGADT7-PA8 into the yeast strain AH109 and activation of reporter genes was monitored by using quadruple synthetic dropout media and α-X-gal according to the instructions of the Matchmaker Yeast-2-Hybrid system (BD Biosciences). As positive controls, pGBKT7-PrP23–231 co-transformed with PA8 and pGBKT7-p53 with pGADT7-SV40 large T-antigen, respectively, were used. pGBKT7-PrP23–231 co-transformed with pGADT7-trxA served as a negative control.

### Modeling of the PrP100–120-PA8 Interaction

To investigate the interaction between PA8 and PrP100–120, protein-protein docking was applied to screen the potential amino acids that play a critical role in forming the complex. A homology model for PA8 was built using the automated mode in SWISSMODEL [46, 47] based on the PA8 sequence [42] and with PDB id 2O8V chain B [48] as template. Second, PDB file 2KUN ([49]; human PrP^C^), an NMR structure that includes the region PrP100–120 for 20 conformations, was obtained from Protein Data Bank. The region PrP100–120 was extracted from all 20 conformations. The two amino acid substitutions between human and mouse in this region (M109L and M112V) were modified in all conformations in 2KUN-PrP100–120 using SWISS PDB Viewer [47] to display the mouse sequence for PrP100–120 in the 2KUN conformations. Third, with the PA8 model as receptor and the 20 (mouse-like) 2KUN-PrP100–120 conformations as ligands, Patchdock [50] was run to create 20 transformations with the lowest energy as starting conformations to be used in the docking step. Docking of the ligand to the receptor was performed using FiberDock [51, 52] which performs flexible refinement and rescoring of rigid body protein-protein docking. FiberDock provided 100 different solutions for each transformation, resulting in a dataset with 2000 theoretical models of receptor-ligand complexes. The complexes where 2KUN-PrP100–120 interacted with PA8 were sorted by global energy score that serves as an approximation of the binding free energy function [51, 52]. From the 50 complexes with the lowest energy, the interaction interface residues from PA8 and PrP100–120 were identified as residues within 4 and 6 Å, respectively. The hydrogen bond formation positions were determined. For residues in PA8 at a distance of 4–6 Å from 2KUN-PrP100–120 that did not make hydrogen bonds, amino acid replacements with potential to improve hydrogen bonding, and consequently to improve the interaction, were identified. Three amino acid replacements were made in the PA8 sequence and homology models were built with the automated mode of SwissModel for each new sequence based on 2O8V [48] chain B and 1TXX chain A [53]. The top three conformations from the first round (PDB id 2KUN, models 6, 9, and 15) for (mouse-like) 2KUN-PrP100–120 were docked to each model using Patchdock and Fiberdock as described above.

### Site-Directed Mutagenesis

PA8 inserted into pQE30 was used as a template for site directed mutagenesis. Partially, complementary oligodeoxyribonucleotide primers of around 40 nucleotides were designed. The reverse primer was common for all the reactions whereas the forward ones differed, carrying point mutations in the non-overlapping region. PCR conditions for 50 μl reactions were as follows: 5 μl 10× Pfu polymerase buffer, 1 μl dNTP mix (10 mM), 1 μl 5′ primer (10 mM), 1 μl 3′ primer (10 mM), and 1 μl PfuUltra High-Fidelity DNA polymerase (Agilent Technologies). One hundred nanograms of plasmid DNA were used as a template. PCR cycling conditions were as follows: 95 °C for 3 min, followed by 30 cycles of denaturation at 95 °C for 30 s, annealing at 65 °C for 30 s and extension at 72 °C for 1.5 min, then final elongation at 72 °C for 3 min. PCR products were purified using QIAquick PCR purification kit according to the manufacturer’s instructions (Qiagen) and then digested with DpnI (New England BioLabs Inc.) in order to eliminate the template. The DNA was then used to transform XL1-Blue chemically competent cells (Agilent Technologies) and amplified. Mutations were confirmed by DNA sequencing (University of Calgary Core DNA Services).

### Recombinant Protein Expression and Purification

*E. coli* thioredoxin A (trxA) and PAs were cloned into pQE30 (Qiagen) as fusion to a N-terminal poly-histidine(6His) tag and then co-transformed with pREP4 into BL21-Gold(DE3) pLysS chemically competent cells (Agilent Technologies). Expression, purification, and refolding of proteins were done as described earlier [42]. After dialysis, protein concentrations were determined by BCA assay (Thermo Fisher Scientific) and adjusted to 3 mg/ml by size exclusion chromatography (Amicon centrifugal filter units, EDM Millipore). Protein purity was assessed by SDS-PAGE (12.5%) followed by Coomassie blue staining.

### Cell Lines and Treatment

Cell lines used in this study were murine neuroblastoma cells N2a (obtained from ATCC; CCL-131) and neuronal CAD5 [54] cells, respectively, not infected or permanently infected with 22L or RML prions, and mouse embryonic fibroblast (MEF) cells uninfected or persistently infected with ME7 prions. N2a-wt cells [9] are N2a stably overexpressing murine PrP^C^, and 3F4-N2a and RML-N2a [55] cells stably overexpress murine PrP containing the epitope for mAb 3F4 and were established by our group. N2a cells were cultured in Opti-MEM (Invitrogen) containing 10% (*v*/*v*) fetal bovine serum (FBS; PAA) and penicillin/streptomycin. CAD5 cells were grown in Opti-MEM with 10% bovine growth serum (Hyclone). MEF cells were kept in MEM (Invitrogen) with the addition of 10% (*v*/*v*) fetal bovine serum and penicillin/streptomycin. All cells were grown at 37 °C in a 5% CO_2_ atmosphere. Purified PAs were added to the culture media at different concentrations and various durations. Fresh PAs were added with each media change which was done every other day. When indicated 10 mM NH_4_Cl was added to the culture media 24 h before lysis. STI571 (Sigma Aldrich) was dissolved in DMSO at a stock concentration of 10 mM.

### Cell Lysis, PK Digestion, and Immunoblot

Postnuclear cell lysis, proteinase K (PK) digestion, and immunoblot were done as described [55]. Briefly, cell cultures were washed with ice cold PBS and then incubated for 10 min with 1 ml ice cold lysis buffer (10 mM Tris-HCL pH 7.5, 100 mM NaCl, 10 mM EDTA, 0.5% Triton X-100, 0.5% sodium deoxycholate). Supernatants obtained upon 1 min centrifugation at 14,000 rpm were used for PK digestion (20 μg/ml PK; 30 min; 37 °C) or PNGaseF deglycosylation. PK digestion was stopped by addition of Pefabloc protease inhibitor, and then, proteins were precipitated by adding 5 volumes of methanol and overnight incubation at − 20 °C. Protein precipitates were resuspended in TNE buffer (Tris-Cl 10 mM pH 7.5, NaCl 150 mM, EDTA 1 mM), loading buffer was added, samples were boiled for 5 min, and aliquots were subjected to SDS-PAGE and immunoblot as described [55] using monoclonal antibodies (mAbs) 4H11or 3F4 for detection of PrP; β-actin (loading control) was detected using an anti-actin mAb (Sigma).

### PNGaseF Deglycosylation

Proteins were methanol precipitated overnight at − 20 °C, then resuspended in a denaturing buffer containing 5% SDS and boiled at 95 °C for 5 min. After chilling them on ice, NP-40 was added to the mixtures to counteract the SDS inhibition of the PNGaseF (New England BioLabs Inc.) activity. The deglycosylation was then carried out at 37 °C for 2 h. Subsequently, a protease inhibitor was added to the samples and the proteins were methanol precipitated overnight at − 20 °C.

### FACS Analysis

Fluorescence-activated cell sorting (FACS) analysis was used to detect cell surface PrP^C^ in N2a cells treated for 4 days with PAs at a concentration of 25 μg/ml. Cells were detached from the plates using 1 mM EDTA (Sigma-Aldrich), centrifuged, and resuspended in fresh FACS buffer (2.5% FCS in PBS) for 10 min for blocking. Then, they were incubated with the mAb 4H11 primary antibody (1:100) or pAb 531 for 30 min, washed three times with FACS buffer, and incubated with the goat anti-rabbit DyLight 488 IgG secondary antibody (Vector Laboratories; 1:200) for another 30 min. All steps were performed on ice and with cold solutions. After being washed, the cells were fixed with 1% paraformaldehyde. Analysis was performed at the University of Calgary Flow Cytometry core facility. Monoclonal anti-PrP antibody 4H11 has been used previously by us to detect N-terminal deletion mutants of PrP (lacking aa 23–121 [56]) as well as PrP^Sc^ indicating the presence of C-terminal epitopes which might be conformational since in our hands peptide epitope mapping did not identify specific linear epitopes. Polyclonal antibody 531 was developed by our laboratory upon immunization of rabbits with recombinant dimeric PrP [57] and epitope mapping revealed that it recognizes N-terminal epitopes.

### Trypsin Digestion of Cells

N2a-wt cells were treated with PA or not and either lysed directly or digested with trypsin/EDTA (0.25%/1 mM) for 8 min on ice or not. After stopping trypsin digestion with soybean trypsin inhibitor (Sigma) postnuclear lysis and PNGaseF digestion as described for immunoblot was performed.

### Indirect Immunofluorescence Assay and Confocal Microscopy

N2a-wt cells were treated for 3 days with PAs. After treatment, cells were washed once with PBS and then were incubated with extracellular buffer (ECB; 150 mM NaCl, 5 mM KCl, 2.5 mM CaCl_2_·2H_2_O, 1 mM MgCl_2_·6H_2_O, 10 mM HEPES, 10 mM D-glucose pH 7.4) for 5 min at 4 °C. Cells were incubated in primary antibody (anti-PrP antibody, 4H11–1:100 in ECB) for 30 min at 4 °C. Cells were washed with PBS and fixed with 4% paraformaldehyde at RT for 30 min. Cells were blocked in 5% FBS in PBS for 30 min at RT. Fixed cells were incubated with secondary antibody (Molecular probe, Alexa Fluor 555–1:500) and DAPI (Molecular Probes, 1:5000). Then, cells were washed with PBS and mounted. Images were taken using a Zeiss LSM700 laser scanning microscope.

### Statistical Analysis

Immunoblot signals were quantified using Image J or ImageQuant (GE Healthcare) software, for quantification of cell surface PrP levels by FACS analysis mean fluorescence values were used for statistical evaluation. All values were expressed as percentage of the control value (100%). For comparison of multiple groups, one-way ANOVA followed by post hoc analysis with Dunnett’s multiple comparison test was used. For pairwise comparisons, nonparametric Mann-Whitney tests were used. Statistical analysis was done using GraphPad Prism software.

## Results

Our group has previously demonstrated that combinatorial PAs binding to PrP^C^ can be used to interfere with prion conversion in infected cultured cells [42]. We described three PAs that inhibited PrP^Sc^ propagation and which maintained their affinity for PrP^C^ even upon expression within the secretory pathway.

### In Silico Studies Identify Amino Acid Substitutions to Improve Binding of PA8 to the PrP^C^ Central Domain

**Fig. 1:**
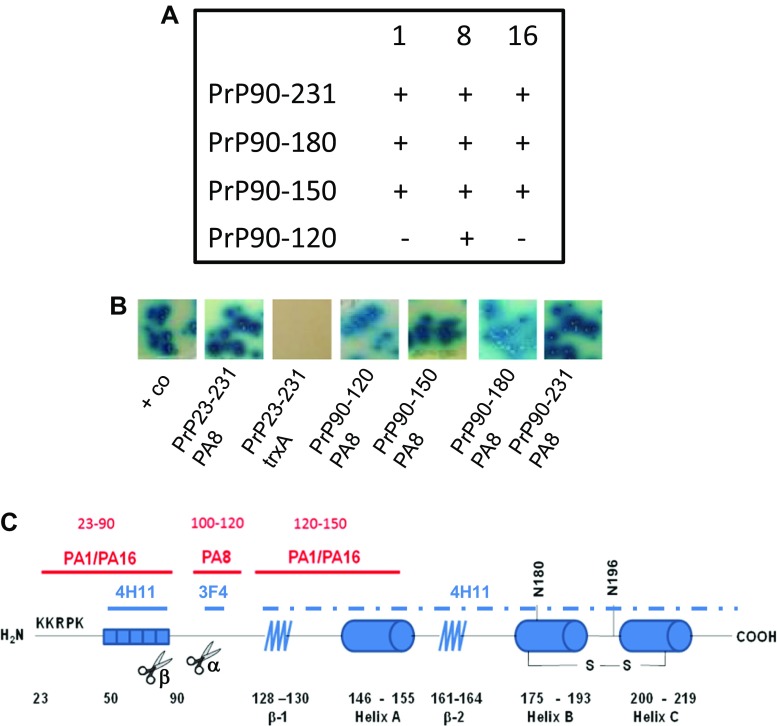
Mapping of PA8 binding sites. **a** Binding sites of PA1, PA8, and PA16 were mapped by yeast-2-hybrid assays using constructs encoding truncated PrPs as bait. It is shown which bait-prey combinations resulted in reporter gene activation, indicative of interaction. **b** Representative result of the mapping experiment for PA8 is shown. As a positive control, pGBKT7-p53 was cotransformed with pGADT7-SV40 large T-Ag. Yeast colonies plated on quadruplicate synthetic dropout medium are shown. Growth of yeast colonies surrounded by a blue corona indicates interaction between bait and prey. **c** Binding sites of the different PAs are summarized and aligned to a model of PrP highlighting secondary structure elements, N-terminal basic and octapeptide repeat regions, α- and β-cleavage sites (aa 110/111 and aa 89/90, respectively) as well as C-terminal posttranslational modifications such as N-linked glycosylation and disulfide bond formation. Epitopes of monoclonal antibodies 3F4 (aa 109–112) and 4H11 are shown, and the dashed line indicates an undefined and nonlinear epitope in the C-terminal part of PrP between amino acids 122 and 231 [56]

First, we aimed to improve the affinity of PAs for PrP^C^. In order to map the binding sites of our previously described anti-PrP PAs which we have shown to interact with either PrP23–100 and/or PrP90–231 [42], we produced truncated versions of PrP by introducing stop codons at amino acids 120, 150 and 180 in PrP90–231. These PrPs were used as baits in a yeast-2-hybrid assay with PA1, PA8, or PA16 as a prey. PA1 and PA16 both interacted with the same PrP C-terminal domains, in addition to the N-terminus. However, PA8 only interacted with the PrP^C^ charge cluster and hydrophobic domain (aa 100–120; summarized in Fig. 1). Representative results of the yeast-2-hybrid assays analyzing the interaction of PA8 with N- and C-terminally truncated PrP are shown in Fig. 1b. For PA8, we identified only one binding site in PrP which simplifies modeling. Moreover, this site has been demonstrated to be critical for prion conversion and reported to be involved in a toxic interaction between PrP^C^ and Aβ oligomers [58]. Therefore, we decided to employ PA8 in modeling studies with the goal to improve the PrP-PA8 interaction.

**Fig. 2:**
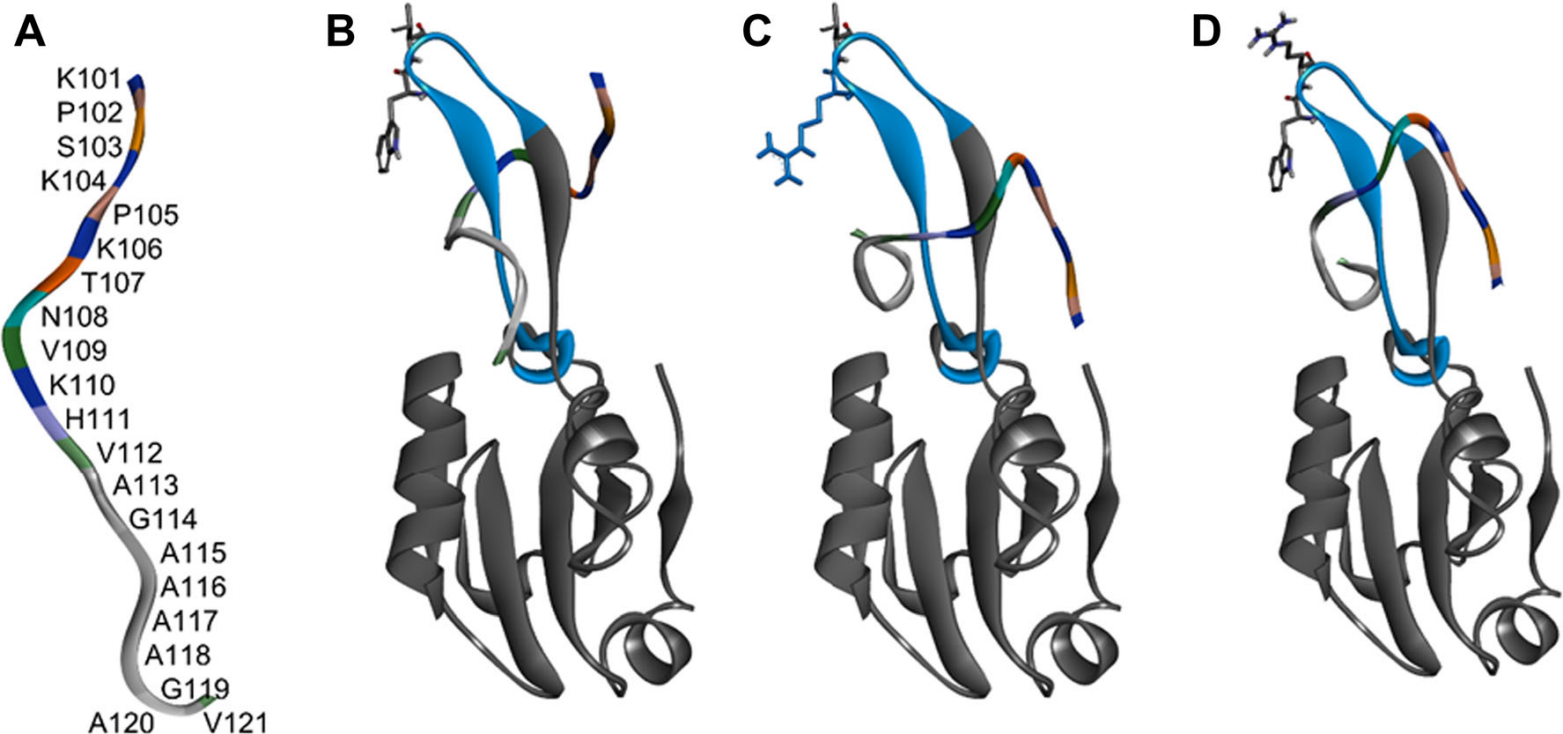
Prion peptide docked to PA8-thioredoxin. **a** One of 20 starting conformations for mouse PrP 101–121 used for docking is shown, colored by amino acid identity. **b** One example of the initial docking of PA8 (blue) inserted into thioredoxin A (gray) and PrP colored by amino acid as in **a**. W46 and V47 (shown as sticks) are within 4–6 Å of PrP. **c**, **d** Top scoring examples of PA8 W46R and PA8 V47R after the second docking round. The mutated positions (W46R and V47R) are shown as sticks. For additional information see supplementary material

We performed in silico docking studies to model the PrP100–120-PA8 complex and to identify PA8 nonhydrogen bonding residues within a distance of 4–6 Å to the PrP moiety (Fig. 2). Residues W46, V47, and T51 (numbering including trxA backbone) fit these criteria, and our theoretical model suggested that replacing these residues with amino acids with longer polar side chains could lead to a stronger interaction between PA8 and PrP^C^ by enabling additional hydrogen bonds to form between the aptamer and the PrP fragment (Fig. 2). Additional docking studies, with PA8 in silico mutated as single mutants of W46R, V47R, and T51R, revealed that the top ten complexes for the mutants were similar to the PA8 complex in the global energy score used by Fiberdock [51, 52] to rank docking solutions (Supp. Fig. 1). The complexes for PA8 and mutated PA8 showed high diversity in specific interactions formed between the different PA8 mutants and the PrP fragment (Supp. Fig. 2). The diversity in interactions is expected since the PrP fragment is dynamic and truncated in both the N-terminus and the C-terminus. Based on the docking results for the in silico mutations, we generated single mutants of PA8 by selectively exchanging the original amino acids at positions 46, 47, and 51 by site-directed mutagenesis. Tryptophan at position 46 was substituted with arginine (46R), lysine (46K), glutamine (46Q), and tyrosine (46Y). The following valine was replaced with arginine (47R) and histidine (47H) and at residue 51 arginine (51R) and lysine (51 K) were introduced instead of threonine (Table 1).

**Table 1:** Primary structures of PA8 and its variants

Peptide aptamer	Amino acid sequence
PA8	ARFEYLRDGY **WV** WRF **T**
PA8-46 K	ARFEYLRDGY **K** VWRFT
PA8-46Q	ARFEYLRDGY **Q** VWRFT
PA8-46R	ARFEYLRDGY **R** VWRFT
PA8-46Y	ARFEYLRDGY **Y** VWRFT
PA8-47H	ARFEYLRDGYW **H** WRFT
PA8-47R	ARFEYLRDGYW **R** WRFT
PA8-51 K	ARFEYLRDGYWVWRF **K**
PA8-51R	ARFEYLRDGYWVWRF **R**

Residues of PA8 in bold were targeted and exchanged by amino acids depicted in red

### Improved Anti-Prion Effects of PA8 Mutants

**Fig. 3:**
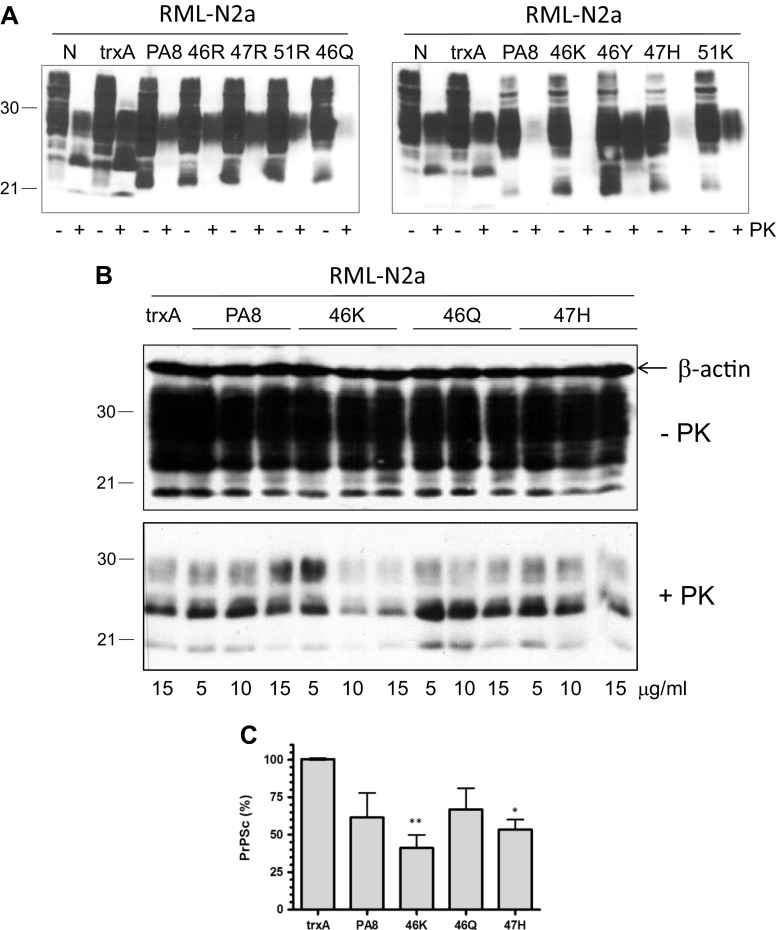
Anti-prion effects of PA8 variants. RML-N2a cells were treated with PA8 and its variants at a concentration of 25 μg/ml (**a**) or with increasing concentrations of PA8, 46K, 46Q and 47H (**b**) for 4 days. Cells were lysed, digested with PK or not as indicated and PrP was detected by immunoblot using mAb 4H11. Nontreated cells (N) and cells treated with thioredoxin A (trxA) served as negative controls, and β-actin was used as a loading control. **c** PrP^Sc^ signals upon treatment with 15 μg/ml of five independent experiments (*n* = 5) as described in **b** were quantified (ImageQuant TL). Signals observed in cells treated with trxA were set as 100%, and all other values were expressed as percentage thereof. Statistical analysis was performed using one-way ANOVA test followed by post hoc analysis with Dunnett’s multiple comparisons. *p value < 0.05; **p value < 0.01. Bars represent average ± standard error of mean (SEM)

In order to verify the anti-prion activity of the newly generated Pas, we expressed them in *E. coli* and purified the recombinant PA8 and its mutants utilizing a 6xHis tag fused to the N-terminus of the PAs. We used these proteins to treat N2a cells infected with scrapie strain RML (RML-N2a). The treatment was performed for 4 days at a nontoxic concentration of 25 μg/ml (assessed by MTT assay; Supp. Fig. 3); then, the cells were lysed and lysates were subjected to proteinase K (PK) digestion or not. An aliquot of each sample was analyzed by immunoblot, and the amount of PK-resistant PrP^Sc^ was compared among the treatments. Untreated cells and cells treated with the scaffold protein thioredoxin A (trxA)-treated cells were used as negative controls. TrxA which does not interact with PrP^C^ did not reduce PrP^Sc^ levels. We found that three of the newly generated PAs (46K, 46Q, and 47H) had an improved activity in reducing PrP^Sc^ levels than PA8 (Fig. 3a). Next, we wanted to confirm an improvement of anti-prion activity of the three PA8 mutants by studying the dose response of PrP^Sc^ reduction. Therefore, we treated RML-N2a cells with PA8, 46K, 46Q, and 47H at increasing concentrations (5, 10, or 15 μg/ml for 4 days). Nontreated cells and trxA-treated cells were used as controls. PrP^Sc^ levels were measured by immunoblot analysis of PK-digested cell lysates. In addition, we verified the amount of PrP in the lysates without PK digestion and used β-actin as a loading control (Fig. 3b) in order to confirm that the observed reduction of PrP^Sc^ was not a result of unequal loading or reduced PrP^C^ levels in PA treated cells. Quantification of the PrP^Sc^ signals in cells treated with 15 μg/ml and statistical analysis revealed that only 46K and 47H significantly reduced PrP^Sc^, with 46K showing a more pronounced effect (Fig. 3c).

**Fig. 4:**
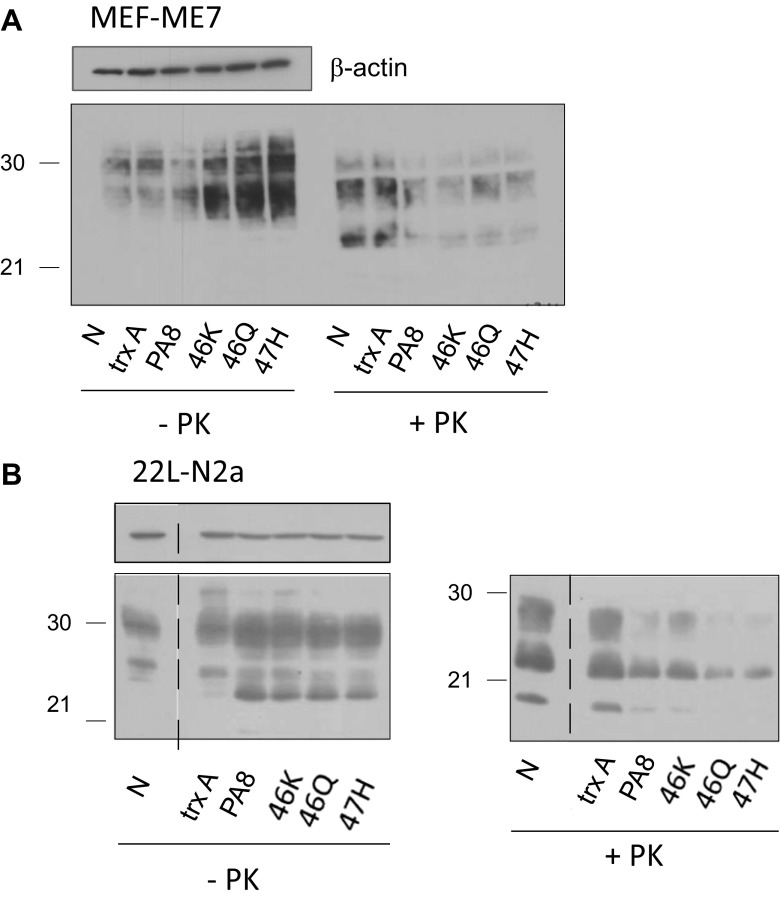
Effects of PA8 and its variants on PrP^Sc^ levels are independent of the prion strain. MEF-ME7 (**a**) or 22L–N2a (**b**) cells were treated for 4 days with 25 μg/ml trxA, PA8, or PA8 variants 46K, 46Q, and 47H, respectively, or not treated (N) as indicated. Upon lysis, aliquots of samples treated with PK (+PK) or not (−PK) were analyzed by immunoblot using anti-PrP antibody 4H11. As a loading control, immunoblots without PK digestion were reprobed with anti-β-actin antibody. The dashed lines in **b** indicate a cut in the immunoblot image which contained unrelated samples that were loaded between the nontreated control (N) and the trxA- or PA-treated samples

When evaluating anti-prion compounds, it is critical to test whether a reduction of PrP^Sc^ can be obtained for different prion strains. Therefore, we analyzed inhibition of propagation of different mouse-adapted scrapie prion strains. Moreover, we wanted to verify any possible influence of the cell line on the anti-prion effect triggered by the PAs. For these purposes, we used PA8, 46K, 46Q, and 47H at a concentration of 25 μg/ml to treat MEFs persistently infected with ME7 prions (MEF-ME7; Fig. 4a) and 22L-infected N2a cells (22L-N2a; Fig. 4b). Again, untreated cells and cells treated with trxA were used as controls. Immunoblot analysis of PK digested cell lysates indicated that the PA’s strong anti-prion activity is independent of the prion strains and the cell lines used. Again, the reduction of PrP^Sc^ in both cell lines was not the result of reduced PrP^C^ levels (Fig. 4; −PK). In order to verify the long-term effect of PA treatment on PrP^Sc^ levels, we added trxA, PA8, and the PA8 mutants (25 μg/ml) for 10 days to the culture medium of RML-N2a cells. In addition, the drug STI571 (10 μM; [59]) served as a positive control and standard for comparison; nontreated cells were used as a negative control. After the treatment period, the compounds were withdrawn and cells were lysed or cultivated further for 5 days without treatment (Supp. Fig. 4). Samples without and with PK treatment were analyzed by immunoblot for PrP content, β-actin served as a loading control. After 10 days of treatment, PrP^Sc^ signals were strongly reduced at similar levels in STI571- and PA8-treated cells, respectively. Strikingly, all PA8 mutants had a more pronounced inhibitory effect on PrP^Sc^, with 46K resulting in a reduction to almost undetectable levels. After 5 days without drug treatment, PrP^Sc^ content in all cell lysates were higher than immediately after treatment, but still reduced when compared to the trxA- or non-treated RML-N2a cells (Supp. Fig. 4).

**Fig. 5:**
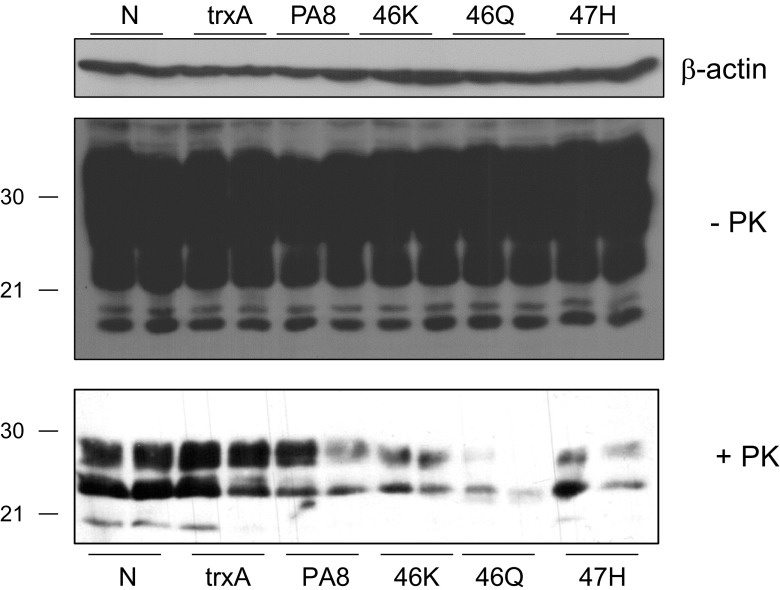
Inhibition of new infection by PA treatment. N2a-3F4 cells (in duplicates) were pretreated for 24 h with PAs or trxA or left untreated (N). Then RML-infected brain homogenate (0.1%) was added for 24 h while PA treatment was continued. Upon removal of brain homogenate and PAs, cells were cultivated and analyzed after passage 5 for PrP^Sc^ accumulation by immunoblot analysis of PK-digested cell lysates using mAb 4H11. In addition, aliquots of samples without PK digestions were analyzed using 4H11 for detection of PrP or anti-β-actin to control for equal loading. Additional replicates of the experiment are shown in the supplemental material

Next, we compared the potential of PA8 and the mutants to interfere with de novo prion infection of N2a cells. We used a highly susceptible clone of 3F4-N2a cells and incubated the cells with trxA, PA8, 46K, 46Q, 47H, or with no PA for 24 h. Then, RML-infected brain homogenate was added for 24 h and incubated in presence of the PAs. After removal of brain homogenate and PAs, the cells were cultivated further without treatment, and passage 5 was tested for PrP^Sc^ accumulation. Whereas in non- or trxA-treated cells a strong signal of PK-resistant PrP was detectable, less PrP^Sc^ signal was observed in PA-treated cells which was not due to an overall reduction of PrP^C^ levels (Fig. 5). This experiment was repeated several times and allowed to reproduce the inhibitory effect of all used PAs (Supp. Fig. 5). However, low infection efficiency likely due to the low dose of brain homogenate (0.1%) used in these experiments required long exposure times of immunoblots which did not allow reliable quantification.

Altogether, these data demonstrate the success of our in silico approach to improve anti-prion effects of PA8. We have generated PA8 mutants which effectively inhibit prion infection of N2a cells and have an improved dose-dependent anti-prion activity compared to the original PA8 in persistently infected N2a cells.

### Peptide Aptamers Promote the Physiological α-Cleavage of PrP^C^

**Fig. 6:**
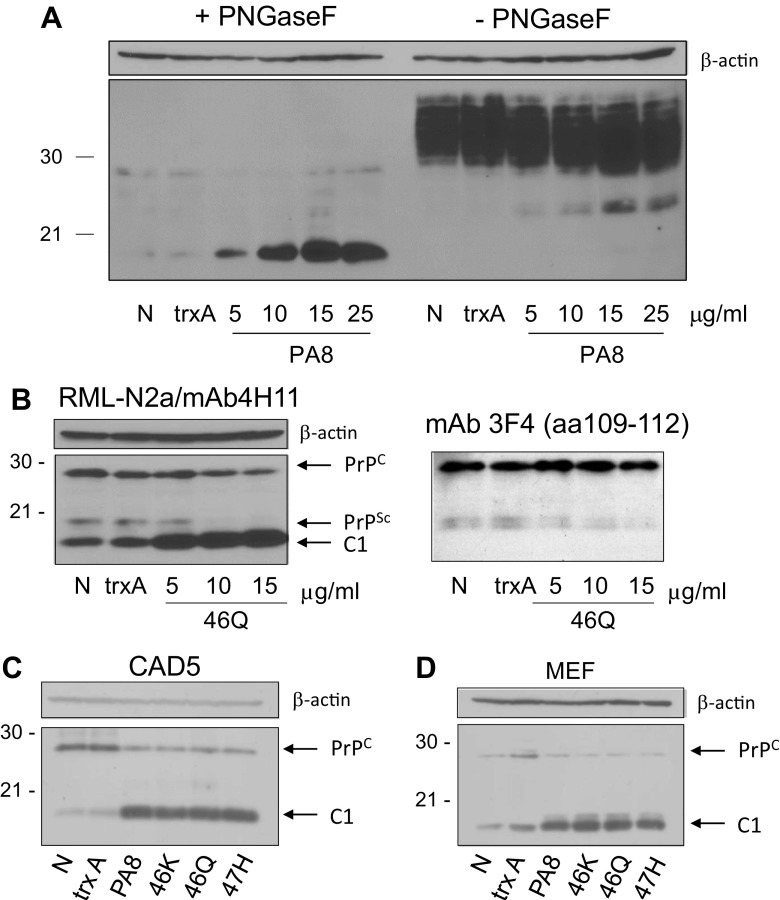
Peptide aptamers enhance the α-cleavage of PrP^C^ independent of prion infection. **a** 3F4-N2a cells were treated for 4 days with increasing concentrations of PA8 as indicated. Cells were lysed and lysates – or + PNGaseF digestion were analyzed by immunoblot using mAb 4H11. Nontreated cells (N) or cells treated with trxA served as negative controls. β-Actin was used to confirm equal loading. **b** RML-N2a cells were treated for 4 days with 46Q as indicated. Lysates were treated with PNGase F and subjected to immunoblot analysis using mAb 4H11 (left panel). The membrane was dehybridized and then incubated with mAb 3F4 (right panel). β-Actin served as a loading control and is valid for both panels. Untreated cells (N) and cells treated with trxA were used as controls. Similar analysis was performed with non-infected CAD5 (**c**) or MEF (**d**) cells which were treated with trxA, PA8, or PA variants for 4 days at 25 μg/ml, or which were not treated (N)

With the previous experiments, we managed to define the binding site of PA8 to PrP^C^, and we achieved to improve its affinity resulting in an improved anti-prion dose-response which is influenced neither by the prion strain nor by the cell line. However, we have noticed an increased strength of the PrP signal in cell lysates without PK digestion treated with PA8, which appears contradictory to inhibition of prion conversion. In order to verify whether this is observed in noninfected cells, we treated uninfected 3F4-N2a cells with increasing concentrations of PA8. Since mainly lower molecular weight and unglycosylated forms of PrP^C^ appeared to be affected, we deglycosylated the lysates of those cells using PNGaseF and compared signals without and with PNGaseF digestion (Fig. 6a). A slight increase of PrP^C^ was observed without PNGaseF digestion despite equal loading. Upon deglycosylation, full-length PrP^C^ (approximately 26 kDa) and an 18-kDa fragment were detectable, presumably corresponding to the C1 fragment of PrP^C^. Next, we treated RML-N2a cells with increasing concentrations of 46Q and deglycosylated samples to confirm that this fragment will be increased by a different PA than PA8 and to investigate the relationship between PrP^Sc^ and C1 fragment abundance. The signal of the band presumably corresponding to C1 was profoundly stronger in lysates of 46Q-treated cells whereas PrP^Sc^ was reduced, demonstrating an inverse correlation of the PrP^C^ fragment and PrP^Sc^ signal intensities (Fig. 6b). It has been known that the C1 fragment is not recognized by the monoclonal anti-PrP antibody 3F4 which recognizes an epitope spanning aa 109–112. Since the RML-N2a cells used in our study express 3F4-PrP^C^ in addition to wild-type mouse PrP^C^, we reanalyzed the same immunoblot after antibody dehybridization with the monoclonal antibody (mAb) 3F4. Whereas full-length PrP^C^ was detectable as well as the decreasing PrP^Sc^ signal, the 18-kDa PrP band was not visible. This supports our assumption that the signal detected by mAb 4H11 corresponds to the C1 fragment of PrP.

The interaction of PAs with PrP^C^ suggests that the increase of the C1 fragment formation induced by PA treatment is independent of prion infection and presumably independent of the cell line that expresses PrP^C^. In order to verify this hypothesis, we investigated the effect of PA treatment on PrP^C^ α-cleavage using different uninfected cell lines. CAD5 and MEF cells were treated with PA8, 46K, 46Q and 47H at 25 μg/ml for 4 days. Then, the cells were lysed and the proteins deglycosylated. From the immunoblots, it was evident that the PA treatment enhanced the C1 fragment formation in comparison to the untreated cells and the cells treated with 25 μg/ml trxA (Figs. 6c, d), independent of the cell type. Notably, these cells only express mouse PrP and the same effect on α-cleavage was achieved as in RML-N2a and 3F4-N2a which in addition to endogenous mouse PrP^C^ express 3F4-tagged mouse PrP, which might influence α-cleavage [60].

**Fig. 7:**
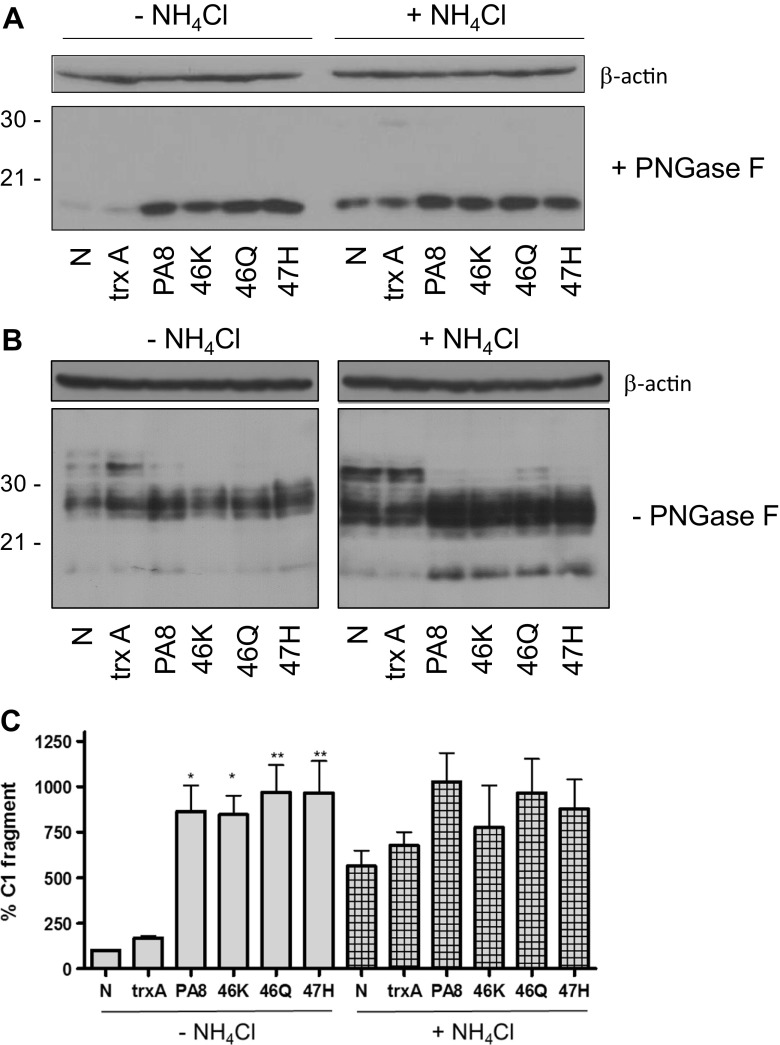
Increased α-cleavage upon PA treatment does not occur in acidic vesicles. Two sets of 3F4-N2a cells were treated for 4 days with PAs at a final concentration of 25 μg/ml. Twenty-four hours before lysis, 10 mM NH_4_Cl was added to the media of one set of cells. Then, cells were lysed, proteins were deglycosylated using PNGase F (**a**) or not (**b**)**.** Aliquots were subjected to immunoblot analysis using mAb 4H11 for detection of PrP and anti-β-actin to demonstrate equal loading. Nontreated cells (N) and cells treated with 25 μg/ml trxA were used as controls. **c** Signals for C1 fragments of three independent experiments (*n* = 3) were quantified. Values of nontreated cells (N) were set as 100% and all other values were expressed as percentage thereof. Statistical analysis was performed using one-way ANOVA followed by post hoc analysis with Dunnett’s multiple comparisons to test the significance of differences between groups without NH_4_Cl and between individual samples with and without NH_4_Cl. *p value < 0.05; **p value < 0.01. Bars represent average ± SEM

To address whether the observed PA-induced α-cleavage of PrP^C^ occurs in acidic vesicles, we tested the activity of PAs in uninfected cells in the absence or presence of ammonium chloride (NH_4_Cl). NH_4_Cl is known to raise the pH in acidic endocytic vesicles thereby inhibiting proteolysis in these compartments [7]. If PA-mediated α-cleavage occurred in such acidic vesicles, treatment with NH_4_Cl would prevent the processing. To address this question, N2a cells were treated in duplicate for 4 days with PAs at a concentration of 25 μg/ml. Twenty-four hours before lysis, NH_4_Cl (10 mM) was added to the media of one set of cells. This treatment efficiently inhibits endolysosomal degradation which we demonstrated previously by analyzing the half-life of epidermal growth factor receptor (EGFR; [61]). Cells were lysed, and the proteins deglycosylated or not. The effect of the treatments in the presence or absence of NH_4_Cl in samples with and without PNGaseF digestion was assessed by immunoblot. Untreated cells and cells treated with 25 μg/ml trxA were used as controls and β-actin served as a control for equal loading. In samples both with and without NH_4_Cl, PA treatment increased the amount of C1 fragment. In lysates of NH_4_Cl treated cells, C1 fragment was increased in non- and trxA-treated cells when compared to lysates of cells without NH_4_Cl. In PA-treated cells, C1 fragment levels were similar to those without NH_4_Cl treatment (Fig. 7a). Due to low amounts of protein loaded after PNGaseF digestion, full-length PrP was barely detectable and only visible upon strong overexposure (Supp. Fig. 6). Levels of PrP^C^ without PNGaseF digestion were similar within groups of cells treated with NH_4_Cl or not (Fig. 7b). In order to assess the significance of our results, we quantified the amount of C1 fragment without and with NH_4_Cl treatment. Statistically significant increased levels of C1 fragment was found in all PA-treated cells when compared to untreated cells (Fig. 7c). However, no statistically significant differences were observed between NH_4_Cl-treated and untreated cells, even though the amount of C1 in nontreated and trxA-treated cells was higher upon NH_4_Cl addition (Fig. 7c).

This suggests that without PA-PrP^C^ interaction the C1 fragment is eventually internalized and degraded in acidic vesicles, whereas in PA-treated cells the degradation of C1 appeared to be inhibited, presumably due to increased impairment of C1 internalization.

In summary, these findings suggest that the α-cleavage in both control and PA-treated cells occurs at or before PrP^C^ reaches the cell surface. Only in control cells the C1 fragment is internalized and degraded in acidic vesicles, whereas in PA-treated cells inhibition of degradation in acidic vesicles does not increase C1.

### PA Treatment Increases Cell Surface Levels of PrP

**Fig. 8:**
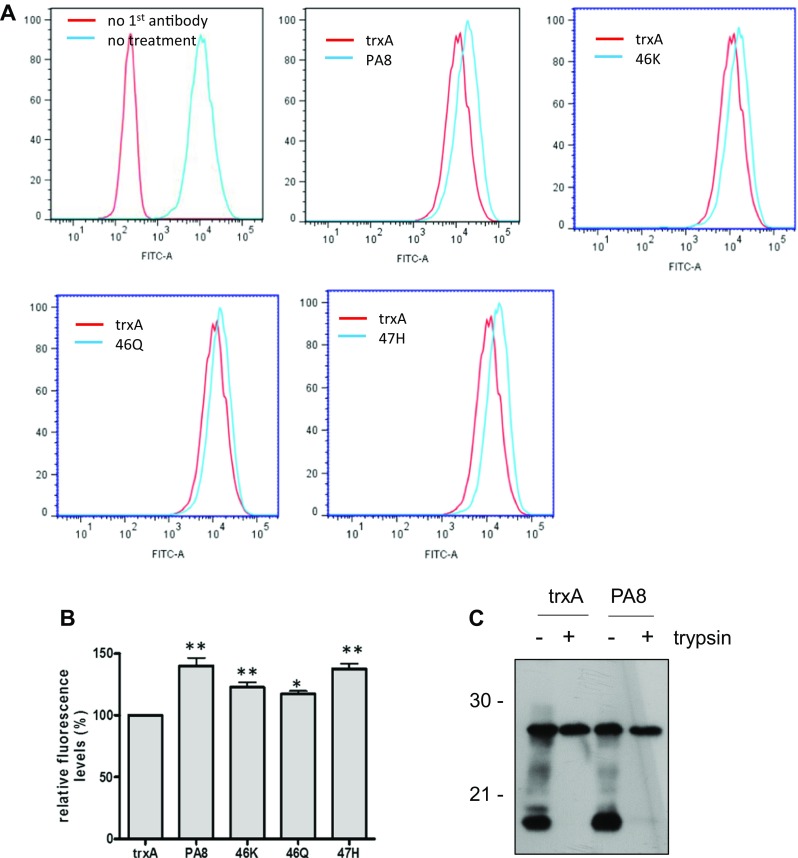

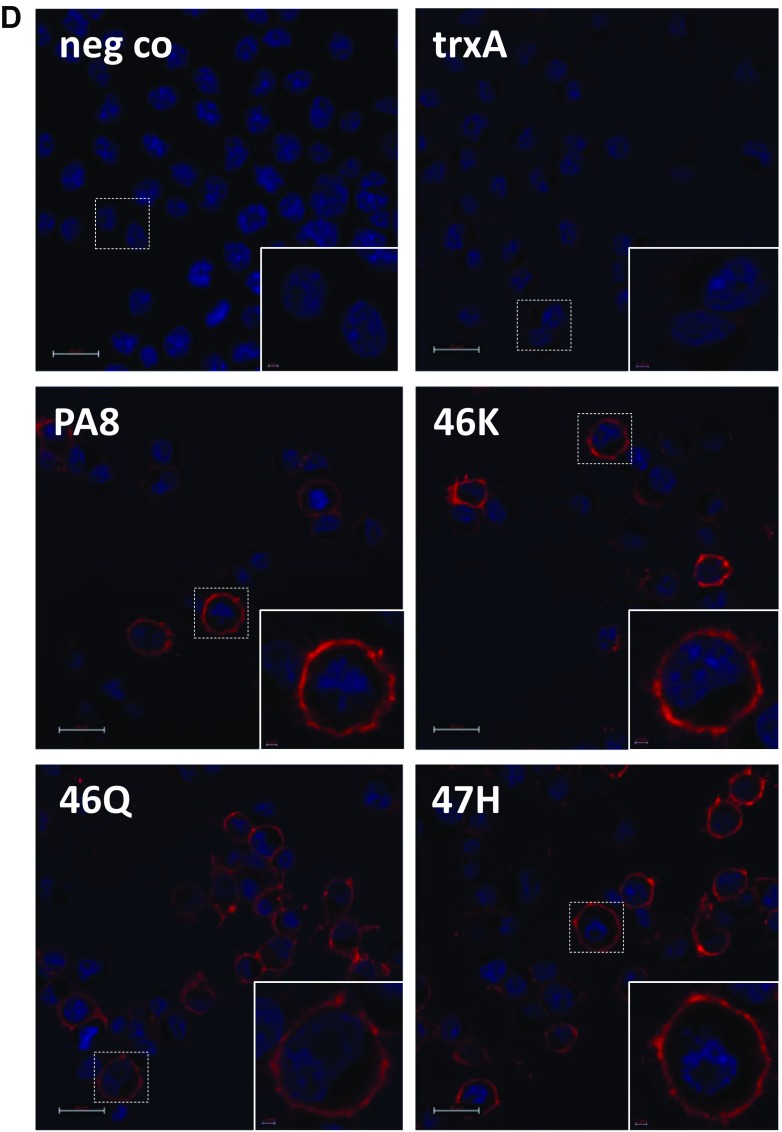
PA treatment increases PrP^C^ levels at the cell surface. **a** N2a cells were treated for 4 days with PAs at a concentration of 25 μg/ml. Levels of cell surface PrP^C^ were analyzed by FACS upon staining with mAb 4H11 and DyLight488-conjugated secondary antibody. As negative control, addition of the primary antibody was omitted. Untreated cells served as positive control. Overlay graphs of trxA-treated cells and cells treated with each of the PAs are shown. **b** Mean fluorescence values of PA-treated N2a cells of seven independent experiments (*n* = 7) were compared relatively to the average fluorescence value of trxA-treated N2a cells, which was set to 100%. Statistical analysis was performed by one-way ANOVA and Dunnett’s multiple comparison tests using GraphPad Prism software. *p value < 0.05; **p value < 0.01. Bars represent average ± SEM. **c** N2a-wt cells were treated with trxA or PA8 for 2 days at 25 μg/ml. Cells were digested with trypsin or not prior to lysis and lysates subjected to PNGase F digestion. Aliquots were analyzed by immunoblot using 4H11. **d** N2a-wt cells without treatment (neg co) or upon trxA or PA treatment (25 μg/ml for 2 days) were incubated on ice with mAb 4H11. Upon fixation, AlexaFluor555-conjugated anti-mouse IgG was added. Nuclei were visualized with DAPI staining. Images were acquired with a Zeiss LSM700 confocal scanning microscope. Scale bar = 20 μm. Inserts show the magnification of a single cell from the image (scale bar = 2 μm)

Our data demonstrate that PA treatment increases PrP^C^ levels, enhances α-cleavage of PrP^C^ at the cell surface or before it reaches the plasma membrane and possibly interferes with internalization of PrP^C^ and/or the C1 fragment. Therefore, we investigated the levels of PrP^C^ specifically at the cell surface by performing FACS analysis in nonpermeabilized and noninfected cells. N2a cells were treated for 4 days with PAs at a concentration of 25 μg/ml. TrxA-treated cells were used as control, and the levels of cell surface PrP^C^ in PA-treated cells were compared to the control cells. Figure 8a shows a right-shift of the graph depicting fluorescence intensity and indicative of the amount of cell surface PrP, in all cells treated with either of the PAs. We compared the average mean fluorescence values of PA-treated cells to trxA-treated cells. Statistical analysis revealed a consistent and statistically significant increase of cell surface PrP levels in all PA-treated cells (Fig. 8b). Preliminary data indicate that this increase was not evident when an anti-PrP antibody was used which recognizes N-terminal epitopes (Supp. Fig. 7).

To further verify the localisation of the C1 fragment, we treated N2a-wt cells with trxA or PA8 for 3 days and then performed a mild trypsin digestion to remove cell surface proteins. Following lysis and deglycosylation with PNGaseF, immunoblot analysis revealed that after trypsin digestion the C1 fragment was undetectable in trxA-treated cells and showed only a very weak band in PA8 treated cells (Fig. 8c), confirming that the vast majority of C1 is located at the cell surface. An overall increase of PrP^C^ cell surface localization was further confirmed by confocal microscopy analysis of N2a-wt cells treated with PAs or trxA as a control (Fig. 8d).

Altogether, we demonstrate that the anti-prion effect of PA8 in persistently infected cells was improved upon in silico modeling and targeted amino acid substitution. Treatment with PAs that interact with the central hydrophobic domain of PrP^C^ and cover the α-cleavage site increases physiological processing of PrP and higher levels of C1 fragment at the cell surface, presumably by interference with PrP^C^ and/or C1 internalization.

## Discussion

Intensive research over the last three decades has provided invaluable mechanistic insights into PrP^C^ biology and into processes that are required for its conversion into pathological PrP^Sc^. Considerable efforts were made to translate this knowledge into therapeutic approaches. However, to date, no successful treatment strategy for prion diseases has been identified.

The current study is built on the hypothesis that increasing the binding affinity of previously selected PAs [42] that interact with PrP^C^ will enhance their inhibitory effect on prion propagation. Based on binding site mapping which revealed that PA8 only had one binding site in PrP^C^ to a domain that is critically involved in the conversion process and the fact that the PA8 binding site partially covers the major interaction site of Aβ oligomers to PrP^C^ [58], we selected PA8 for further investigations. In a previous approach to improve the binding affinity of PAs targeting cyclin-dependent kinase 2, error-prone PCR was employed for affinity maturation, resulting in a new library of PA variants that were screened by yeast-2-hybrid for high-affinity interactors [62]. As an alternative to affinity maturation by error-prone PCR, we decided to use a more targeted approach to determine mutations in PA8 that may likely increase the binding affinity. By modeling the PrP100–120-PA8 complex in silico, three amino acids (W46, V47, T51; Table 1) were identified that were within a distance of 4–6 Å to PrP100–120. These residues were targeted for substitutions with amino acids that could improve the hydrogen bonding potential of these sites, followed by another round of modeling to confirm which substitution brought PA8 into proximity of PrP100–120 as an indicator of maintained or improved binding affinity (Fig. 2). According to the results of our theoretical model, we synthesized eight new recombinant PAs (Table 1) with a prospective improved binding to PrP^C^. Three of them (46K, 46Q, 47H) were selected for further analysis based on their inhibitory effect on prion propagation. Two of them demonstrated a significantly stronger inhibitory effect on PrP^Sc^ propagation in RML-N2a cells than the original PA8, and all PAs effectively inhibited de novo infection. Anti-prion effects were independent of cell line and prion strain used for infection. In an experiment to determine the long-term effect of PA treatment on PrP^Sc^ levels, none of the used compounds including the previously described STI571 [59] cured RML-N2a cells from infection, however, the PA8 mutants exhibited the most pronounced reduction. The finding that upon STI571 treatment for 10 days the cells were not cleared from PrP^Sc^ is contradictory to an earlier study [59], however, might be related to more sensitive detection tools in immunoblot and a higher infection rate of the cells compared to those previously used.

Interestingly, PA8 binds the cellular prion protein between residues 100 and 120. It is the most conserved part of PrP [44, 45] and crucial for the conversion of PrP^C^ to PrP^Sc^. This region almost completely overlaps with the proposed neurotoxic PrP106–126 peptide [63]. It has been described to have an intrinsic ability to form β-sheet structure in vitro [63] which is the main distinctive feature in the formation of PrP^Sc^ [64]. It enables the interaction of the cellular and infectious PrP isoforms, supports β-sheet formation and lipid interactions and acts as a hinge region in prion conversion [65–68]. Under physiological conditions, this domain is cleaved by an αPrPase between residues 110 and 111 thereby inactivating its neurotoxic potential. This process produces a short soluble N-terminal fragment, termed N1, and a GPI-anchored fragment, named C1 [69]. Some evidence indicated that processing is mediated by ADAM10 for constitutive cleavage and ADAM17 activity upon stimulation by agonists of the protein kinase C pathway [16, 23]. However, recent studies argue against an involvement of ADAM10 in the constitutive cleavage [22, 24, 70]. In addition, PrP^C^ is cleaved by a β-secretase around residues 89/90 as a response to oxidative stress, leading to the formation of a shorter N-terminal peptide (N2) and of a GPI-anchored fragment (C2) that is prone to undergo conformational changes [69, 71]. Structural stabilization of the N-terminal octapeptide repeat region mimicking a state with four Cu^2+^ ions bound to PrP enhances β-cleavage [72]. Our findings that PA interaction with PrP^C^ enhanced αPrPase cleavage might be similarly related to structural stabilization of the flexible N-terminal part of PrP^C^. Upon PA treatment, this flexible part of PrP could be locked in a conformation that can serve as an ideal substrate for αPrPase and thereby be cleaved in a more efficient manner. Notably, homo-dimerization of PrP^C^ as a physiological stress response or induced PrP^C^ dimerization which may have similar effects as PA binding causes enhanced α-cleavage [26, 73].

We also observed an increase in cell surface levels of PrP^C^ in PA-treated cells which can be a result of significantly increased C1 fragment formation. As the N-terminal basic amino acids are critical for internalization [74] the increased α-cleavage and the higher ratio of C1 fragment which lacks the basic motif results in a decreased internalization rate and accumulation of C1 at the cell surface [38, 75]. Whereas in untreated cells the C1 signal increases upon NH_4_Cl treatment indicating its eventual degradation in acidic vesicles, this is not observed in PA-treated cells. Therefore, we argue that the PA interaction with PrP^C^ exaggerates inhibition of PrP^C^ and/or C1 internalization. In our 3F4-N2a cells, we observed a high baseline level of α-cleavage, and such fluctuations have been observed by others even for different batches of the same cell line [39]. The potential C1 accumulation can further explain the high abundance of C1 in PA-treated 3F4-N2a cells and the comparatively low levels of full-length PrP, as there is not only a shift to increased α-cleavage, but in addition potentially a reduced degradation. Nevertheless, our data further show that inhibition of endolysosomal proteolysis by NH_4_Cl treatment does not reduce the C1 fragment formation in PA treated cells. This is in agreement with α-cleavage at the plasma membrane or the late secretory pathway [7, 25, 75] and indicates that PA binding does not alter the compartment where cleavage occurs.

The C1 fragment cannot be converted to PrP^Sc^ and is a transdominant negative inhibitor of prion conversion. It has been reported that cell lines with naturally high level of α-cleavage have an enhanced resistance to prion infection [39]. In scrapie-challenged mice expressing wild-type PrP, the coexpression of C1 drastically slowed the PrP^Sc^ deposition and extended the incubation period of disease [38]. Moreover, the increased amount of C1 fragment and decreased content of C2 in ovine brain tissue has been associated with resistance to scrapie. It has in fact been suggested that the proteolytic processing of PrP^C^ in this species is likely to be connected to the prion protein genotype. Notably, a recombinant C1 protein derived from the ARR variant, associated with resistance to disease, was able to inhibit fibrillation of the full length PrP [76].

Taken together, these findings suggest that PAs have a dual effect on prion conversion: (i) inhibition of the PrP^C^-PrP^Sc^ interaction and (ii) increased formation of C1 fragment. The impact on α-cleavage appears to be similar for all PAs we analyzed. These observations can serve as an explanation for the seeming discrepancy of the effects of PAs in persistently infected cells versus de novo infection. In persistently infected cells, PAs compete with PrP^Sc^ for binding to PrP^C^; therefore, a higher affinity will make them more potent to inhibit prion conversion and differences can be observed for the PAs. In de novo infection, cells were pretreated with PAs which bind to PrP^C^ non-competitively thereby increasing α-cleavage and abundance of C1 and reducing full-length PrP which is eligible for conversion. Since α-cleavage is similarly enhanced by all PAs in non-infected cells, the impact on de novo infection is expected to be similar for the different PAs under our experimental conditions.

Moreover, an enhanced α-cleavage can be beneficial in other conditions not related to prion infection. Interestingly, PA8 and its variants target a PrP^C^ domain which does not only overlap with the α-cleavage site but also partially covers the binding site of toxic Aβ oligomers. It has been demonstrated that Aβ42 interacts with PrP95–105, which functions as a receptor to mediate deleterious effect exerted by Aβ oligomers [58]. Moreover, the N1 fragment protects primary cultured neurons against Aβ oligomer-associated toxicity [77]. We speculate that the partial overlap of the PA-PrP^C^ and the Aβ oligomer-PrP^C^ binding interfaces may be sufficient for competitive inhibition of Aβ oligomer toxicity and impairment of long term potentiation upon PA treatment. In addition, the increased α-cleavage not only affects the levels of C1, but presumably also the amount of the protective N1 fragments. This suggests that our PAs interacting with PrP^C^ are potentially beneficial against Aβ induced effects by two mechanisms, namely by inhibition of interaction and by increasing the levels of the neuroprotective N1 fragment.

We present here that the anti-prion activity of PAs can be enhanced by in silico modeling and targeted mutagenesis. We show a novel approach to enhance protective α-cleavage of PrP^C^ by a mechanism that does not require the knowledge which protease mediates the processing, and occurs upon PA interaction with the hydrophobic domain. In light of the tight connection between Alzheimer’s and prion disease pathologies, compounds such as our PAs that target PrP100–120 and are able to modulate the protective posttranslational cleavage of PrP^C^ might have remarkable effects on Aβ oligomer toxicity or neuroprotection, as presumably also the levels of the N1 fragment increase. Our future studies will aim towards structure-based drug design [41] to identify small molecules mimicking the PA-PrP^C^ interaction in order to be able to apply the concept in vivo. Overall, we suggest that the PAs described in this study can be used as a basis for identification of drugs that potentially can be applied for the treatment of prion and other neurodegenerative diseases that benefit from inhibition of toxic or enhancement of protective functions of PrP or PrP fragments.

## Electronic supplementary material

ESM 1 (PDF 835 kb)
