# Supplementary material for: Interaction of Peptide Aptamers with Prion Protein Central Domain Promotes α-Cleavage of PrPC
Source: Mol Neurobiol. 2018 Feb 19;55(10):7758–74. doi: 10.1007/s12035-018-0944-9 (PMC6132731; doi:10.1007/s12035-018-0944-9)
Supplement: Supplementary file 1 — (PDF 835 kb) [file 12035_2018_944_MOESM1_ESM.pdf]

Supp. Fig. 1

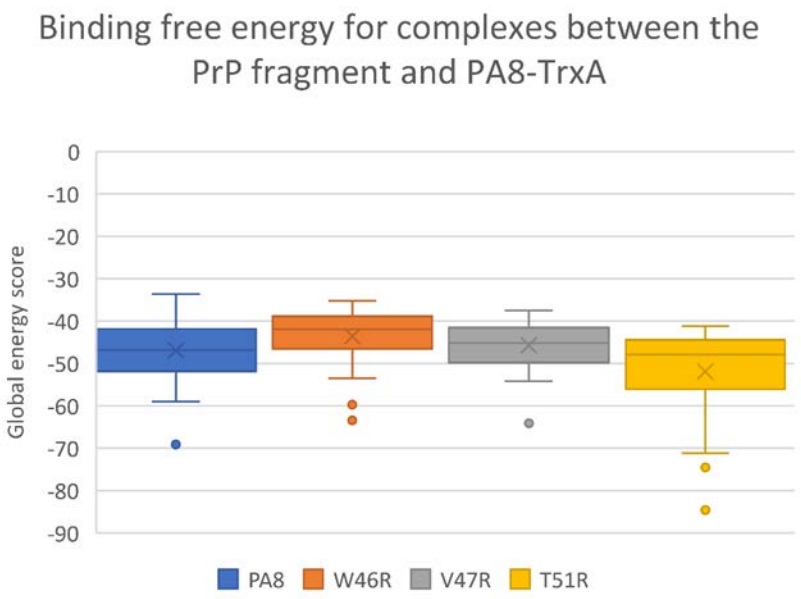
Binding free energy for complexes between the PrP fragment and PA8-TrxA
Global energy score
0
-10
-20
-30
-40
-50
-60
-70
-80
-90
PA8
W46R
V47R
T51R

Supp. Fig. 2

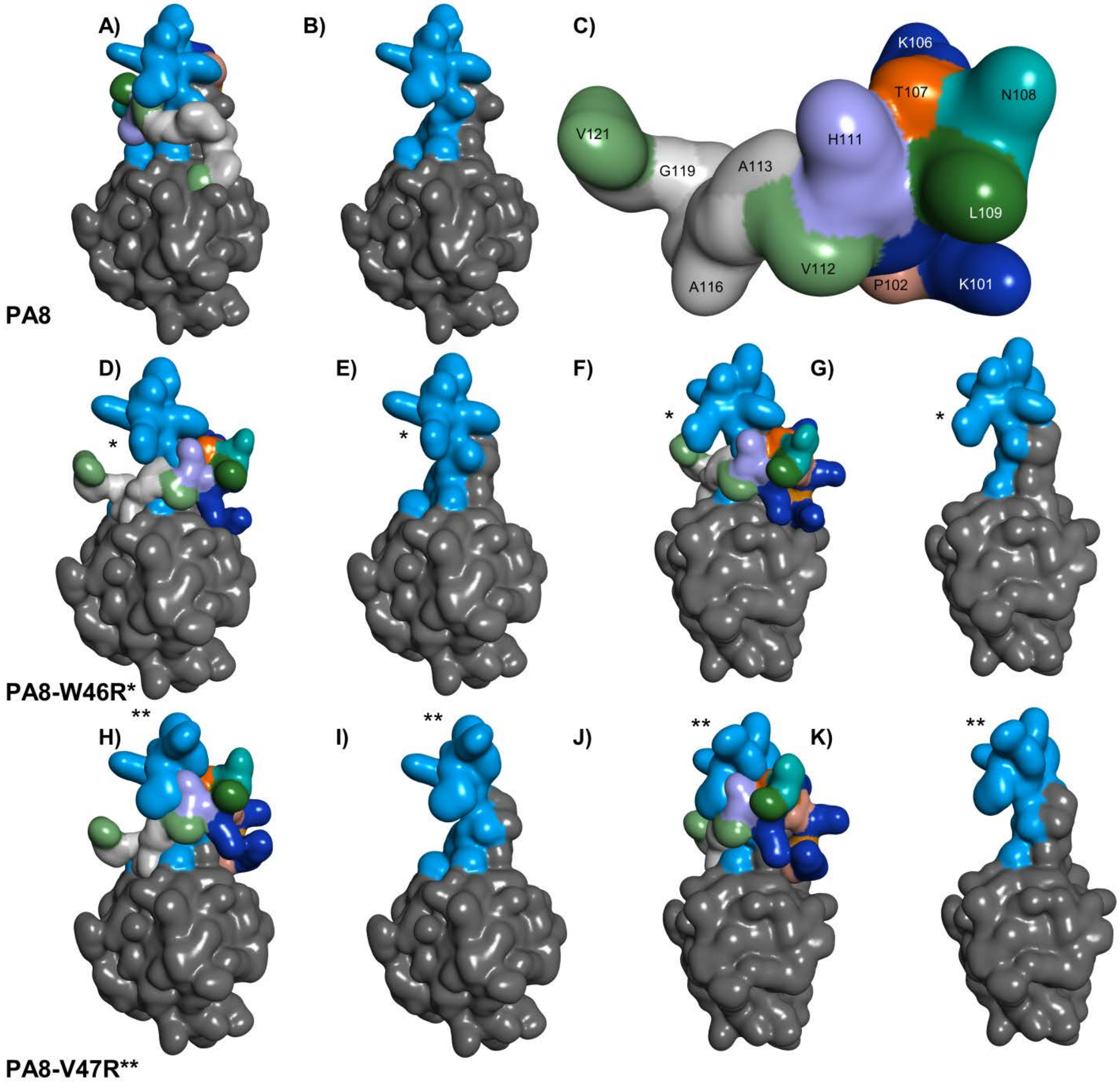
A)
B)
C)
K106
T107
N108
V121
H111
G119
A113
L109
V112
A116
P102
K101
PA8
D)
E)
F)
G)
*
PA8-W46R*
**
H)
I)
J)
K)
PA8-V47R**

Supp. Fig. 3

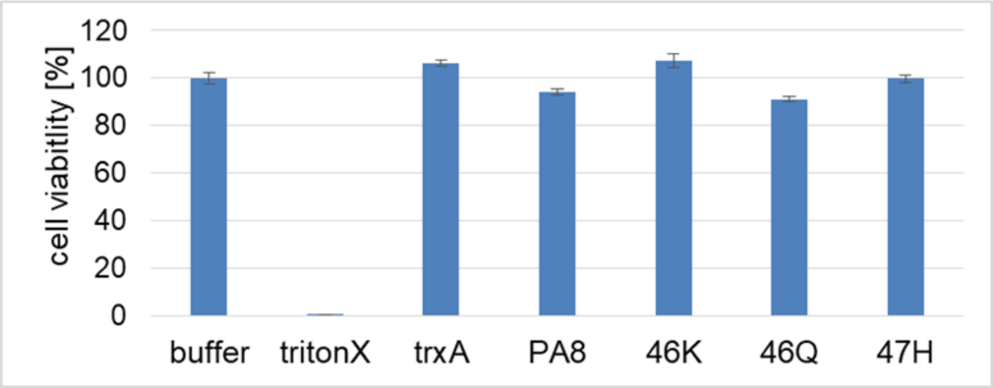

cell viabitlity [%]
120
100
80
60
40
20
0
buffer
tritonX
trxA
PA8
46K
46Q
47H

Supp. Fig. 4

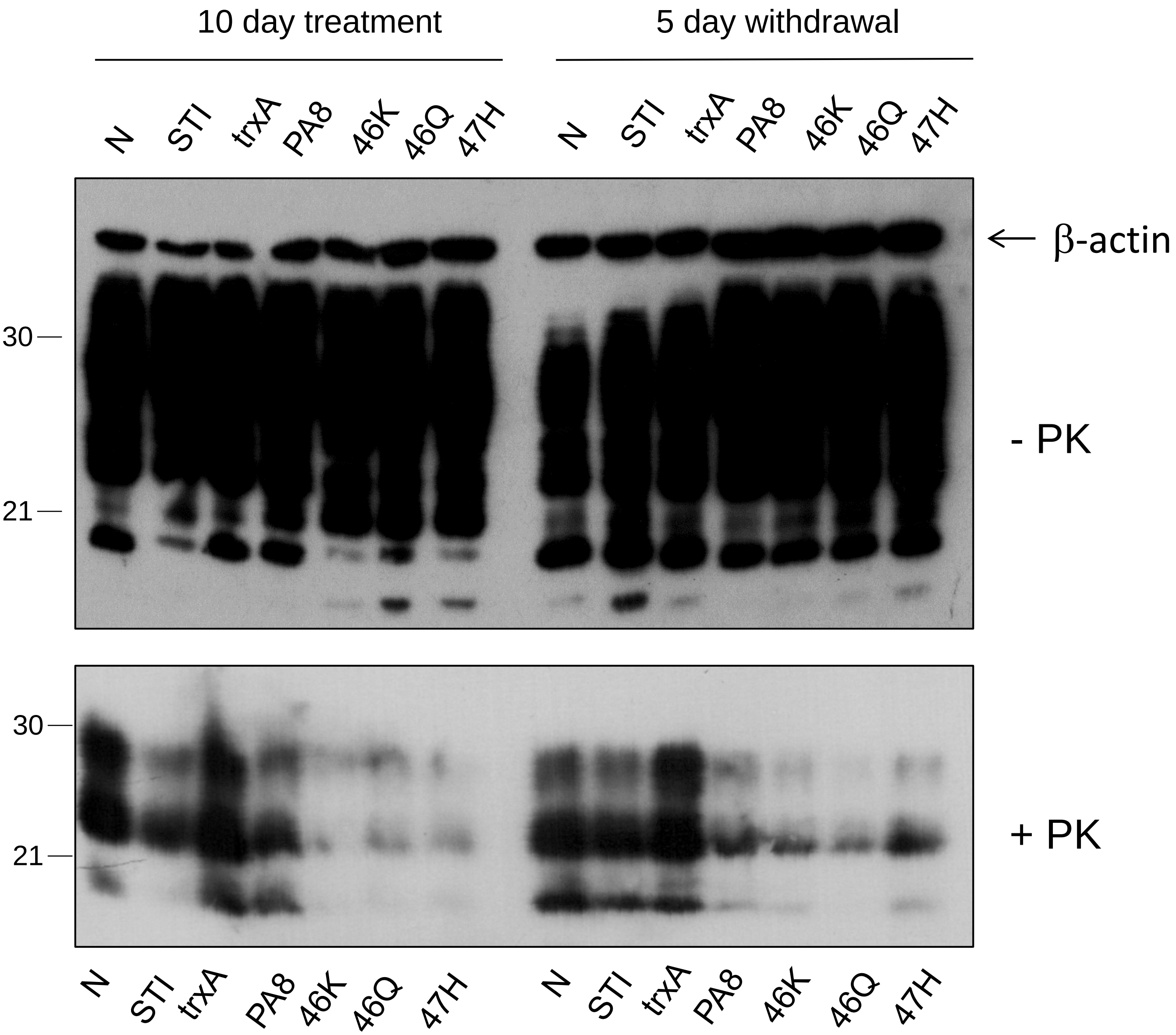
10 day treatment
5 day withdrawal
N
STI
trxA
PA8
46K
46Q
47H
N
STI
trxA
PA8
46K
46Q
47H
← β-actin
30
21
- PK
30
21
+ PK
N
STI
trxA
PA8
46K
46Q
47H
N
STI
trxA
PA8
46K
46Q
47H

Supp. Fig. 5

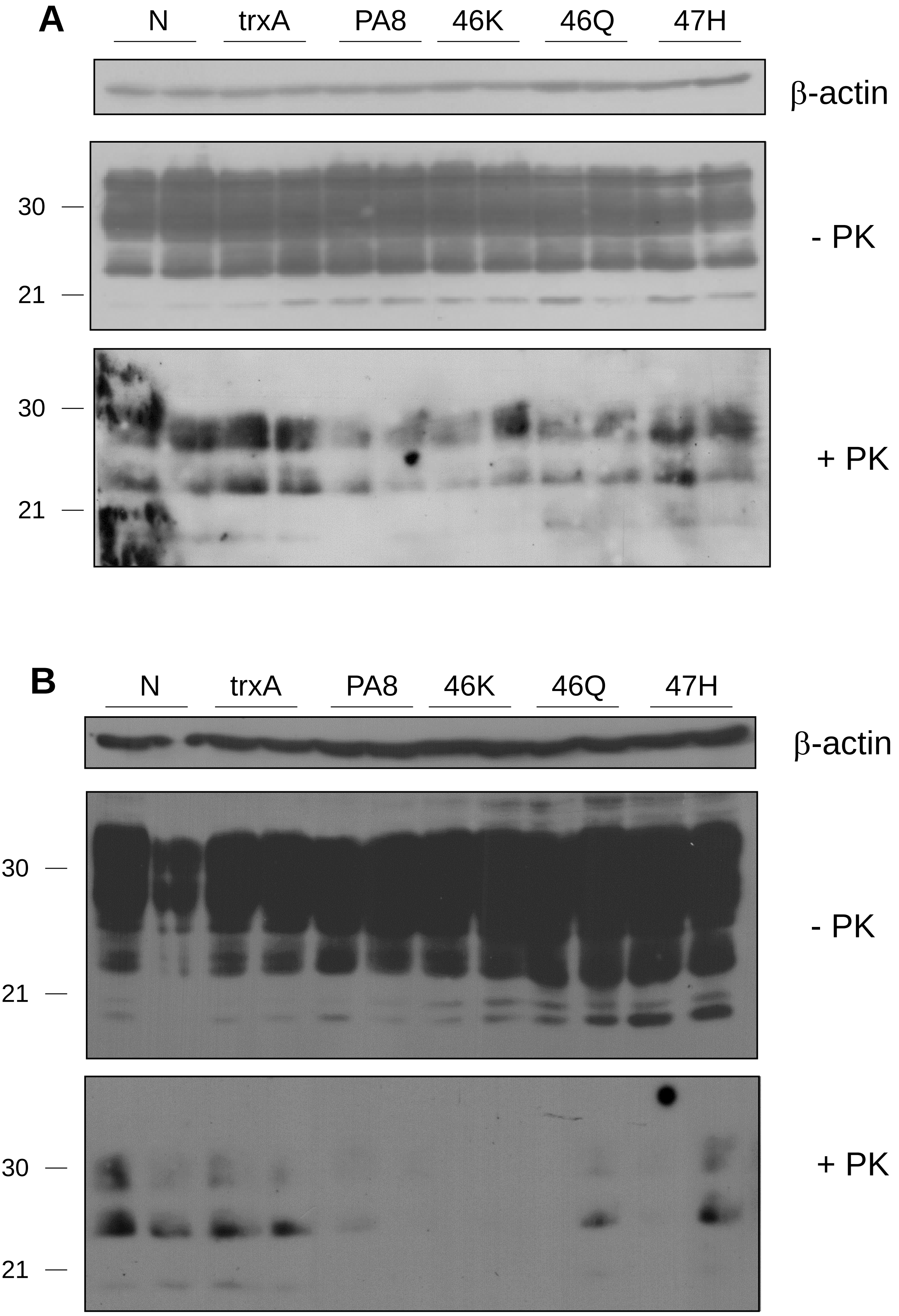

A
N
trxA
PA8
46K
46Q
47H
β-actin
30
21
- PK
30
21
+ PK
B
N
trxA
PA8
46K
46Q
47H
β-actin
30
21
- PK
30
21
+ PK

Supp. Fig. 6

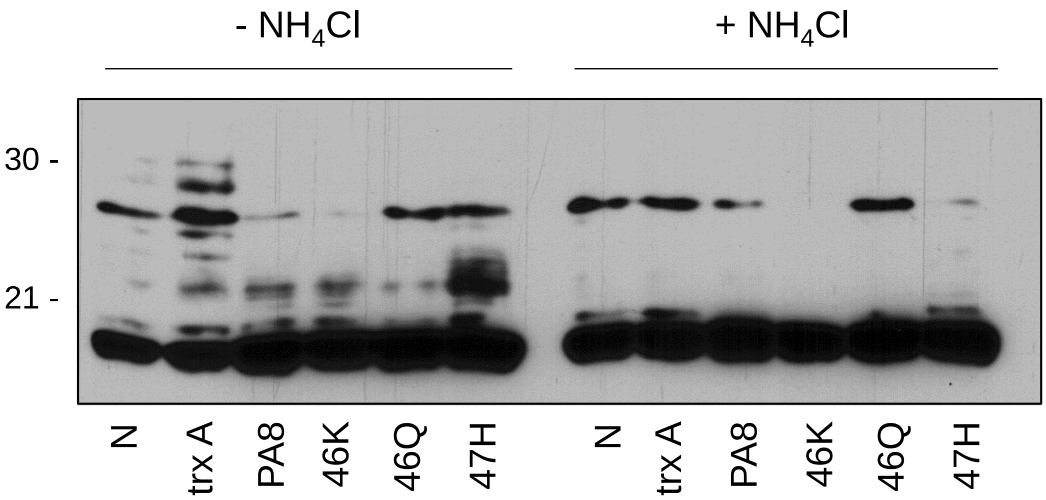

Supp. Fig. 7

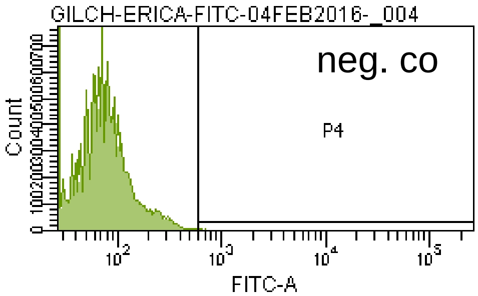

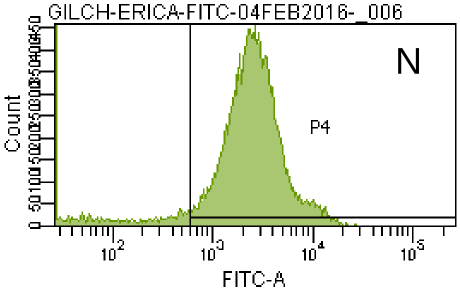

Mean FITC intensity: 3187

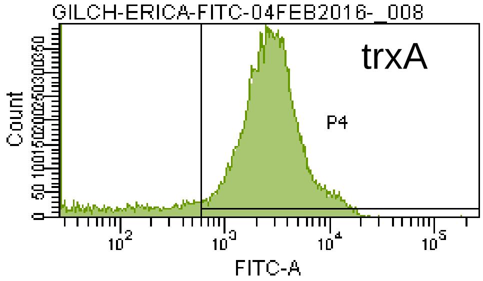

Mean FITC intensity: 3472

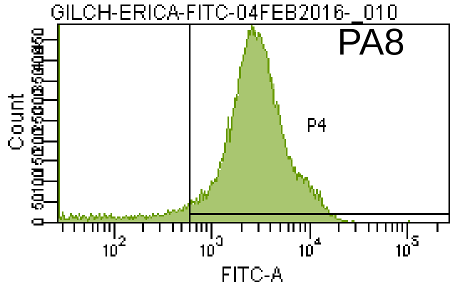

Mean FITC intensity: 3721

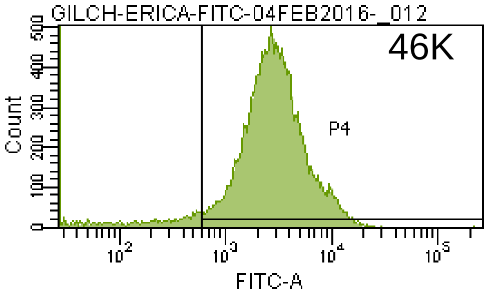

Mean FITC intensity: 3592

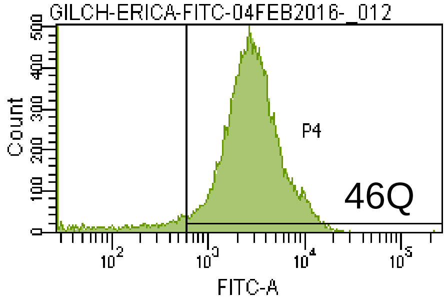

Mean FITC intensity: 3311

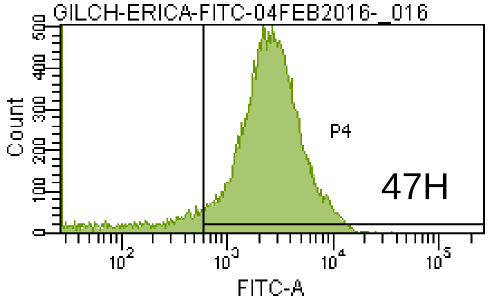

Mean FITC intensity: 3205

Supplement Figure Legends

Supp. Fig 1. Binding free energy for complexes between the PrP fragment and PA8. Analysis of global energy scores in the 10 best docking solutions for the top 3 complexes: 2KUN-M-6, 2KUN-M-9, and 2KUN-M-15. The global energy score is an approximation of the binding free energy used by Fiberdock to rank docking solutions [51,52]. All single mutants have similar variance in global energy score for the top docking solutions, compared to the top 10 PA8-TrxA PrP complexes from the first round. (Mann-Whitney; PA8 vs W46R: *p-value*= 0.055, PA8 vs V47R: *p-value*= 0.495, and PA8 vs T51R: *p-value*= 0.164).

Supp. Fig. 2. Prion peptide docked to PA8. (A) One example of the initial docking of PA8 (blue)inserted into thioredoxin (grey) and PrP colored by amino acid as in **(C)**. W46 and V47 are identified as being within 4-6 Angstrom of PrP. Both shown as surface representations. **(B)** Same as A without PrP bound. **(C)** Surface representation of one of 20 starting conformations for Mouse PrP 101-121 used for docking. **(D, H)** Top scoring examples of PA8 W46R and PA8 V47R, respectively, after the second docking round. Both shown as surface representations. **(E, I)** Same as D and H without PrP. **(F, J)** Sideview of D and H. **(G, K)** Same as **(F)** and **(J)** without PrP. The PrP peptide is in all cases colored as in **(C)**.

Supp. Fig. 3. PA treatment is not toxic. N2a-wt cells were seeded into 96well plates and were treated for 4 days with PAs at a concentration of 25 µg/ml. TrxA and dialysis buffer treatment were used as negative controls, triton-X100 treatment was employed as a positive control. Then an MTT assay was performed according to the manufacturer’s protocol. Cell viability is expressed as percentage of the viability in dialysis buffer treated cells. In all conditions, cell viability was > 92%.

Supp. Fig. 4. Effect of long-term treatment of RML-N2a cells with PAs on PrP^{Sc} levels. RML-N2a cells were treated for 10 days with PAs or trxA at 25 μg/ml, with STI571 (Sigma) at 10 μM or left untreated (N). Cells were either lysed immediately after finishing treatment (10 day treatment) or cultivated further for 5 days without treatment before lysis (5 day withdrawal). All lysates were subjected to PK digestion or not and analysed by immunoblot using mAb 4H11. β-actin was employed as a loading control.

Supp. Fig. 5. Reproducible reduction of *de novo* prion infection upon PA treatment. N2a-3F4 cells (in duplicates) were pre-treated for 24 hours with PAs or trxA or left untreated (N). Then RML-infected brain homogenate (0.1 %) was added for 24 hours while PA treatment was continued. Upon removal of brain homogenate and PAs, cells were cultivated and analysed after passage 5 for PrP^{Sc} accumulation by immunoblot analysis of PK-digested cell lysates using mAb 4H11. In addition, aliquots of samples without PK digestions were analysed using 4H11 for detection of PrP or anti-β-actin to control for equal loading. **(A)** and **(B)** represent results of two independent experiments.

Supp. Fig. 6. Extended exposure reveals full-length PrP after deglycosylation. This figure represents an overexposure of the immunoblot shown in **Figure 7A** to demonstrate the presence of low levels of full-length PrP.

Supp. Fig. 7. Preliminary FACS analysis of surface PrP using polyclonal anti-PrP antibody 531. N2a cells treated for four days with PAs at a concentration of 25 µg/ml. Levels of cell surface PrP^{C} were analyzed by FACS upon staining with pAb 531 and DyLight488-conjugated secondary antibody. As negative control, addition of the primary antibody was omitted. Untreated cells served as positive control. Mean fluorescence values are indicated.
